# Supplementary material for: The causal relationship between the human gut microbiota and pyogenic arthritis: a Mendelian randomization study
Source: Front Cell Infect Microbiol. 2024 Nov 26;14:1452480. doi: 10.3389/fcimb.2024.1452480 (PMC11629706; doi:10.3389/fcimb.2024.1452480)
Supplement: Supplementary file 1 [file DataSheet1.zip › mendelian test/ebi-a-GCST90027673.csv_forest.pdf]

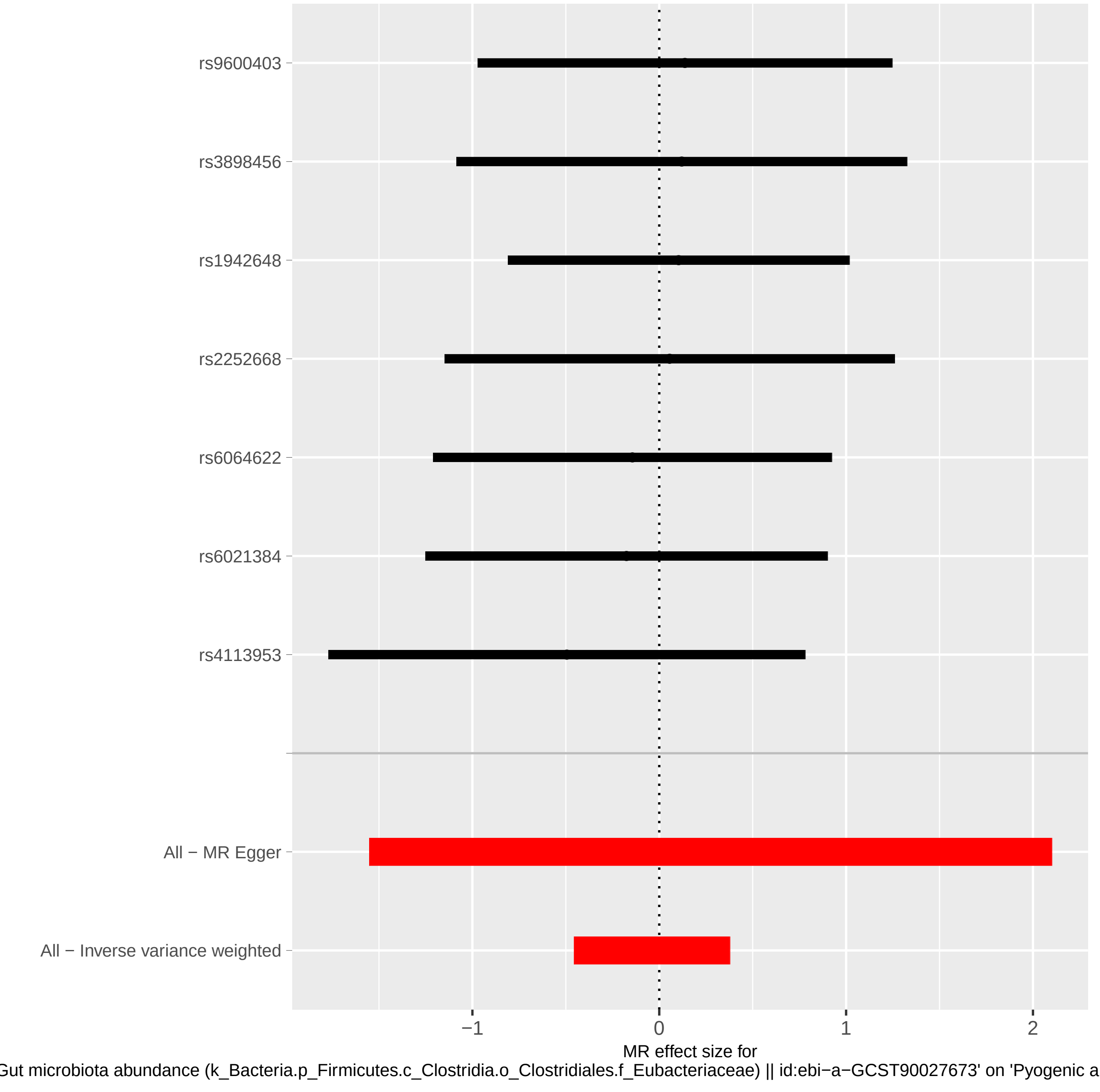
rs9600403
rs3898456
rs1942648
rs2252668
rs6064622
rs6021384
rs4113953
All − MR Egger
All − Inverse variance weighted
−1
0
1
2
MR effect size for
Gut microbiota abundance (k_Bacteria.p_Firmicutes.c_Clostridia.o_Clostridiales.f_Eubacteriaceae) || id:ebi−a−GCST90027673' on 'Pyogenic a
